# Transfer of regulatory T cells into abortion-prone mice promotes the expansion of uterine mast cells and normalizes early pregnancy angiogenesis

**DOI:** 10.1038/srep13938

**Published:** 2015-09-10

**Authors:** Katja Woidacki, Nicole Meyer, Anne Schumacher, Alexandra Goldschmidt, Marcus Maurer, Ana Claudia Zenclussen

**Affiliations:** 1 Experimental Obstetrics and Gynecology, Medical Faculty, Otto-von-Guericke University, Magdeburg, Germany; 2 Department of Dermatology and Allergy, Charité – Universitätsmedizin Berlin, Germany

## Abstract

Implantation of the fertilized egg depends on the coordinated interplay of cells and molecules that prepare the uterus for this important event. In particular, regulatory T cells (Tregs) are key regulators as their ablation hinders implantation by rendering the uterus hostile for the embryo. In addition, the adoptive transfer of Tregs can avoid early abortion in mouse models. However, it is still not defined which mechanisms underlie Treg function during this early period. Cells of the innate immune system have been reported to support implantation, in part by promoting angiogenesis. In particular, uterine mast cells (uMCs) emerge as novel players at the fetal-maternal interface. Here, we studied whether the positive action of Tregs is based on the expansion of uMCs and the promotion of angiogenesis. We observed that abortion-prone mice have insufficient numbers of uMCs that could be corrected by the adoptive transfer of Tregs. This in turn positively influenced the remodeling of spiral arteries and placenta development as well as the levels of soluble fms-like tyrosine kinase 1 (sFlt-1). Our data suggest an interplay between Tregs and uMCs that is relevant for the changes required at the feto-maternal interface for the normal development of pregnancy.

After the egg is fertilized, the uterus prepares for implantation. Genetic factors are relevant for the quality and viability of the blastocyst; however complex interactions between the immune system and the endocrine system are necessary for an adequate environment where the embryo is able to grow. For implantation to be successful, it is necessary to have a healthy uterine milieu that allows the invasion of the blastocyst and the rapid growth of the placenta while supporting the transformation of uterine into decidual cells. This is mediated and facilitated by immune cell populations, the cytokines they secrete and by hormonal changes. Innate immune cells of importance for implantation are present already before pregnancy. They usually present a unique phenotype that greatly differs from the phenotype of their counterparts located in the periphery or in other tissues. Among the innate immune cells, the most intensively studied are the uterine natural killer cells (uNKs)^1^. They contribute to angiogenesis and to the remodeling of spiral arteries (SAs)^1^,^2^. Uterine dendritic cells (uDCs) are present in high numbers at specific sites along the non-pregnant uterus as shown *in vivo*^3^ and are important for implantation^4^,^5^. Uterine mast cells (uMCs) also differ enormously from MCs found in other tissues and are involved in implantation^6^,^7^. Especially MC-associated proteases (Mcpt), including tryptases and chymases, are involved in uMC function^8^. Uterine macrophages directly communicate with trophoblasts in a bidirectional way^9^. They are in charge of maintaining the luteal microvascular network that is necessary for the integrity of the corpus luteum and progesterone production^10^. A recently described population of second-trimester decidual neutrophils shows a unique phenotype and possesses angiogenic properties^11^. From this information, a picture emerges, where cells of the innate immune system are responsible for the changes necessary to support pregnancy, namely the promotion of angiogenesis and tissue remodeling. An imbalance in cell number or abnormal distribution can cause suboptimal angiogenesis, which is often related to pregnancy complications. Elevated levels of the anti-angiogenic factor placental soluble fms-like tyrosine kinase 1 (sFlt-1) has been linked to endothelial dysfunction that in turn causes hypertension and proteinuria in preeclampsia^12^. Angiotensin Receptor II (AT_2_) seems to be of relevance as well as it is involved in cardiovascular functions during pregnancy^13^.

Already at copulation, the female´s immune system gets in contact with male antigens present in sperm; animal studies as well as studies performed with samples from patients immediately after coitus indicate that seminal fluid rapidly attracts cells from the innate and adaptive immune system^14^,^15^. Thus, the maternal immune system is aware very early of the presence of foreign, paternal antigens. Animals lacking T and B cells can get pregnant and have progeny^16^, so that at the first glance it could be inferred that adaptive immune cells are not absolutely necessary for pregnancy. Studies in the last years, however, have shown that a small subtype of adaptive CD4^+^ T cells is necessary for implantation. The depletion of CD4^+^Foxp3^+^ regulatory T cells (Tregs) before mating drastically influences the uterine environment and hampers the implantation of the fetus^17^, while depletion at later time points only provokes a mild augmentation in the abortion rate^18^. Hence, it is of central importance to understand the local mechanisms governing the implantation of the embryo. Here, we used a well-established model in which disturbed pregnancy tolerance can be restored by the addition of Tregs before implantation^19^. With this model, we aimed to elucidate whether Treg transfer activates uMCs and positively influences angiogenesis. Various studies suggest a direct interaction between MCs and Tregs. It could be demonstrated that MCs are critical in Treg-mediated allograft tolerance and that Tregs activate and recruit MCs via IL-9^20^,^21^. The suppression of MC degranulation by Tregs can dampen allergic MC responses, and *in vivo* depletion of Tregs provokes MC-related anaphylactic symptoms^22^,^23^. Furthermore, MC-derived IL-2 supports Treg-mediated suppression during allergic dermatitis^24^ and MC-derived transforming growth factor (TGF-) β is able to induce the conversion of naïve T cells into Tregs *in vitro*^25^. We are particulary interested in the interplay between Tregs and MCs as MCs are not only present in the female reproductive tract^26^,^27^,^28^ but also migrate from the periphery to the uterus upon female hormone augmentation^29^. When pregnancy occurs, uMC numbers further increase and remain high during early gestation^6^. uMCs have a positive influence on placentation and the remodeling of SAs as well as on placenta size and fetal growth. MCs are multifaceted cells that interact with other immune and non-immune cells. Whether Treg/MCs communication or interplay is of relevance for pregnancy establishment and maintenance was not explored and is the main aim of this study.

## Results

### uMC numbers peak during sexual receptivity and remain high if pregnancy takes place in normal but not in abortion-prone mice

**Figure 1:**
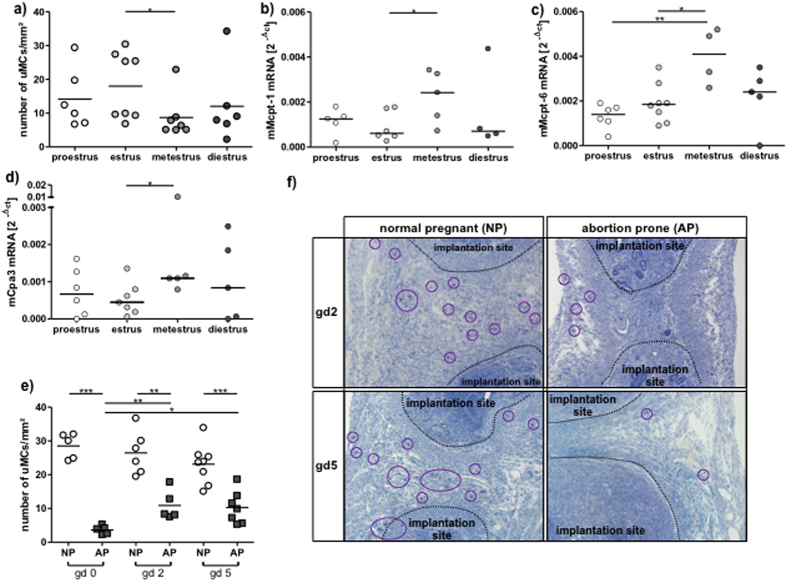
Number of uMCs peaks at sexual receptivity and uMCs expand with the onset of pregnancy. Abortion-prone mice have diminished uMC numbers. (**a**) Number of uMCs per 1 mm^2^ was quantified in Tolouidine blue stained paraffin sections (5 μm) from uteri of virgin CBA/J females (n = 6–8) during different phases of the estrous cycle. The mRNA levels of MC-related proteases Mcpt-1 (**b**), Mcpt-6 (**c**) and Cpa3 (**d**) were measured in uterine tissue of virgin CBA females at proestrus, estrus, metestrus or diestrus. (**e**) uMCs number per 1 mm^2^ was quantified in uterine paraffin sections (5 μm) of BALB/c-paired CBA/J females (normal pregnant, NP) or DBA/2-mated CBA/J females (abortion-prone, AP) at gestational days (gd) 0, 2 and 5 of pregnancy. (**f**) During pregnancy MCs are localized in the decidua between the implantation sites. MCs (magnification ×100) were visualized after Toluidine blue O staining (0.1%) at days 2 and 5 of pregnancy in NP and AP mice and are indicated by circles. For (**a**,**e**) the results are presented as single values with means. Statistical differences were obtained using unpaired t- test (*p < 0.054, **p < 0.005, ***p < 0.001). For (**b**–**d**) the results are presented as individual values plus medians. Statistical differences were obtained using non-parametric Mann-Whitney-*U*- test (*p < 0.054, **p < 0.005).

We first quantified the numbers of MCs in Toluidine blue-stained slides of uteri from CBA/J mice and observed an oscillation in uMC numbers at the different phases of the estrous cycle (Fig. 1a). The number of uMCs peaked at estrus, which represents the period of sexual receptivity. We next studied the mRNA transcripts for molecules that are stored in MC granula and secreted upon activation. mRNA levels of the Mcpt-1, Mcpt-6 and Carboxypeptidase A3 (Cpa3) were significantly elevated at the metestrus phase (Fig. 1b–d). This is interesting as this phase follows the estrus, showing that MC-related proteases augment immediately after the peak of MC numbers, which suggests their local activation followed by the secretion of mediators upon degranulation. This in turn coincides with the time point at which the uterus undergoes degenerative modifications.

We next studied the localization and numbers of uMCs during early pregnancy. For that, we analyzed samples from BALB/c-mated CBA/J females known to have normal pregnancies (NP) and from DBA/2J-mated CBA/J females that have reportedly augmented abortion rates (abortion-prone group, AP) due to impaired feto-maternal tolerance^19^. We did so at days 0, 2 and 5 of pregnancy. We observed that in NP females at all three time points, uMC numbers were even higher than at estrus (Fig. 1a,e). Astonishingly, this was not the case for females from the AP group whose uMC levels were comparable to the ones observed during metestrus (Fig. 1a,e). As for their localization, uMCs were located in close proximity to the developing embryo as shown in Fig. 1f.

### Adoptive transfer of Tregs diminishes the occurrence of fetal death, which is associated with an augmentation of uMCs

**Figure 2:**
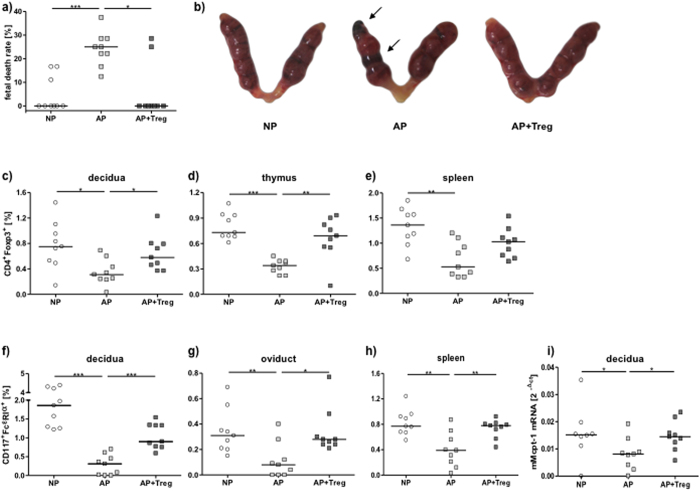
Transfer of Tregs into abortion-prone mice is related with increase of MC numbers and reduction of fetal death rate. **(a)** Fetal death rates were determined at day 12 of pregnancy in normal pregnant (NP, n = 9), abortion-prone (AP, n = 9) and Treg-treated abortion-prone (AP+Treg, n = 9) animals. (**b**) Representative pictures of uteri of NP, AP and AP + Treg are shown. Arrows indicates abortions. The percentage of CD4^+^Foxp3^+^ positive cells in (**c**) decidua (**d**) thymus and (**e**) spleen as well as the percentage of CD117^+^FcεRα^+^ positive cells in (**f**) decidua, (**g**) oviduct and (**h**) spleen was analyzed by flow cytometry at day 12 of pregnancy. (**i**) Mcpt-1 mRNA levels were analyzed by real time PCR and the data expressed as ^2−**Δ**cT^. Data are shown as single values with medians. Statistically significant values are indicated as follows: *p < 0.054, **p < 0.005, ***p < 0.001 (Mann Whitney-*U* test).

We next employed a well-characterized strategy to rescue fetuses from abortion in the CBA/J × DBA/2J combination, which consists in adoptively transferring antigen-specific Tregs immediately after plug detection^19^,^30^,^31^. We confirmed that Treg-transferred females have fewer abortions than the PBS-treated controls and this was comparable with the abortion rate observed in NP animals (Fig. 2a,b). This was associated with augmented CD4^+^Foxp3^+^ Treg numbers in decidua (Fig. 2c), but also in thymus (Fig. 2d) and spleen (Fig. 2e). To evaluate whether Treg transfer has an impact on MC numbers, we studied reproductive tissues as well as the spleen and lymph nodes. The adoptive transfer of Tregs was associated with an augmentation in the proportion of CD117^+^FcεRIα^+^ double positive MCs cells in the decidua, the maternal part of the fetal-maternal interface (Fig. 2f), in the oviduct (Fig. 2g) and in splenic tissue (Fig. 2h) compared to AP mice that were sham-treated. Interestingly, the Treg transfer normalized MC numbers to the levels observed in NP individuals (Fig. 2f–h). In lymph nodes, the MC number remained unchanged (data not shown). Accordingly to the elevated MC numbers, Mcpt-1 mRNA levels in the decidua of AP mice increased after Treg transfer (Fig. 2i). IL-9 mRNA was almost indetectable at the feto-maternal interface but IL-3 and mSCF mRNA were both augmented after Treg transfer (Suppl. Fig. 1). As both factors were proposed to be growth factors for mast cells, it is possible that Treg directly or indirectly augment the levels of both, finally resulting in augmented numbers of uMCs *in situ*. Blockage of IL-10, identified as the main Treg mediator in this particular model^30^, not only abrogated the protective effect of Tregs (see material and methods) but also hindered Mcpt-1 mRNA levels augmentation (Suppl. Fig. 2). As isolation and further culture of uMCs is technically challenging, a final confirmation of functional changes in uMCs remains open, but the augmentation of Mcpt-1 after Treg transfer and its lack of augmentation when the main Treg mediator was blocked strongly suggest that Treg transfer influences not only MC numbers but also their activity.

### Placental development and SA remodeling are impaired in abortion-prone mice and restored by the transfer of Tregs

**Figure 3:**
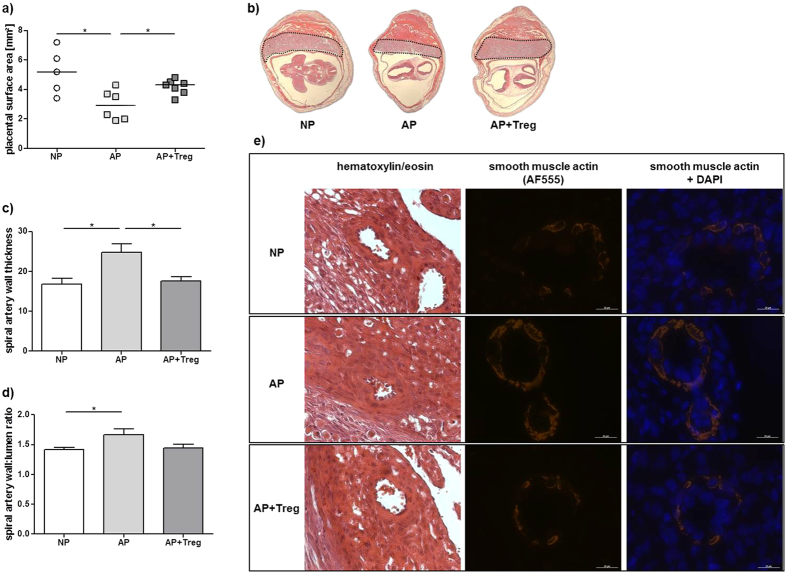
Transfer of Tregs results in elevated numbers of MCs in abortion-prone mice and rescues placental and spiral artery defects. Normal pregnant (NP, n = 5), abortion-prone (AP, n = 6) and abortion-prone animals transferred with Tregs (AP + Treg, n = 7) were sacrificed at day 12 of pregnancy. 5 μm cross sections of paraffin embeeded whole implantation sites (WIS) were stained with H/E. **(a)** Placental surface areas were measured in WIS from NP, AP and AP + Treg mice. The results are presented as single values with medians. 1 placenta was measured for each female and the median was calculated for the group. Statistical differences were obtained using non-parametric Mann-Whitney-*U*- test (*p < 0.054). (**b**) Representative pictures (magnification × 10) of Hematoxylin/Eosin stained sections from NP, AP and AP + Treg mice are shown. Broken lines indicate the placenta areas. Spiral artery (SA) wall and lumen diameters of 2 to 10 SAs per animal of NP (n = 5), AP (n = 6) and AP + Treg (n = 7) were measured and calculated as (**c**) wall thickness and (**d**) wall/lumen ratio. Data are expressed as mean ± SEM and their statistical significance was analyzed by unpaired t-test (*p < 0.054). Representative images of H/E-stained sections (magnification ×400) and immunofluorescence for smooth muscle actin and smooth muscle actin + DAPI (magnification ×1000) in SA of NP, AP and AP + Treg mice are depicted in (**e**) (scale bar = 20 μm).

Next, we studied the placentation and SA remodeling in abortion-prone females. Because MC numbers directly correlate with the remodeling of SAs^6^, we asked whether Treg transfer into abortion-prone females could improve placentation and pregnancy outcome via the upregulation of MC numbers. Placental surface areas were decreased in AP mice, where both Tregs and MCs were present in lower numbers as in NP mice (Fig. 3a,b). This was accompanied by insufficiently remodeled SAs, characterized by an increased wall thickness (Fig. 3c) that resulted in abnormal wall to lumen ratios (Fig. 3d) when compared with NP mice. AP mice showed high SA wall expression of smooth muscle actin and coherently arranged multilayered smooth muscle cells, signs of impaired SA remodeling (Fig. 3e). Adoptive Treg transfer into AP mice completely normalized the shaping of the SAs and resulted in normal placental surface areas; both were comparable to those observed in NP mice (Fig. 3a–e).

### Abortion-prone mice show enhanced expression of placental sFlt-1 and AT_2_, which is reduced by Treg transfer

**Figure 4:**
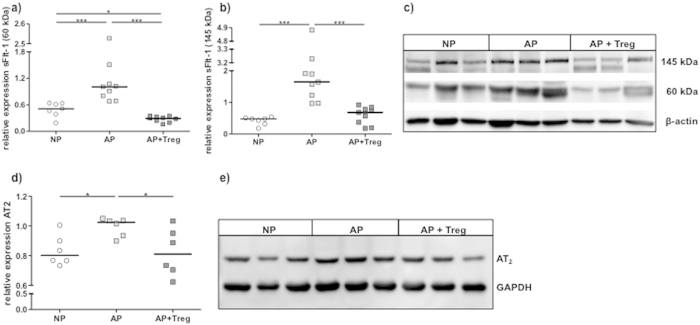
Expression of placental sFlt-1 and AT_2_ in abortion prone mice is normalized after transfer of Tregs. The protein levels of the two pregnancy-relevant isoforms of soluble Flt-1 (**a**) (60 kDa) and (**b**) (145 kDa) as well as (**d**) angiotensin receptor II (AT2) were analyzed in placental tissue of normal pregnant (NP, n = 7), abortion-prone (AP, n = 9) and abortion-prone animals treated with Tregs (AP+Treg, n = 9) by Western blot and expressed as arbitrary units. The data are expressed as medians and analysed by Mann- Whitney-*U* test: p < 0.054, **p < 0.005, ***p < 0.001(Mann-Whitney-*U* test). **(c)** Representative blots for soluble Flt-1 and **(e)** AT2 are shown together with the housekeeping genes beta actin (for sFlt-1) and GAPDH (for AT2).

We next studied the sFlt-1 and AT_2_ expression in placental tissue of AP mice that presented diminished numbers of Tregs and MCs (Fig. 2c–h), smaller placental areas and impaired SA modification (Fig. 3a–e). In placentas from AP mice sFlt-1 was significantly higher expressed when compared to NP mice at pregnancy day 12 (Fig. 4a–c). The adoptive transfer of Tregs, which leads to more uMCs, normalized sFlt-1 expression (Fig. 4a–c). The same was observed for AT_2_ (Fig. 4d,e).

## Discussion

It is widely accepted that major changes at cellular and molecular levels take place in the uterus immediately after fecundation. These dramatic changes allow implantation and support the embryo development and growth. Mouse models are of importance as they can be employed to study the effect of a particular cell type or of cell-cell interactions. Here, we are particularly interested in the possible interaction between Tregs and MCs as both are of pivotal importance for pregnancy albeit their main functions differ greatly. To the best of our knowledge, no studies addressed the possible interaction or interdependence between MCs and Tregs during pregnancy.

Our findings demonstrate that the adoptive transfer of Tregs during early pregnancy influences MCs and this in turn is related to improved SA remodeling and increased placenta size. Interestingly, low levels of Tregs have previously been reported in AP vs NP mice during early pregnancy^19^,^30^,^31^,^32^,^33^. Our results confirm and extend these reports as we also found low numbers of CD4^+^Foxp3^+^ Tregs in AP mice in different immune-related organs as well as in decidua.

Thus, low Treg numbers correlate with low uMC numbers in animals whose pregnancies do not progress optimally. In AP animals, not only a higher fetal death rate could be observed but also a defective placentation. This could explain why Girardi and colleagues observed intrauterine growth restriction (IUGR) in fetuses from this combination^34^. We next transferred Tregs into AP mice. Successful transfer of Tregs was confirmed by detection of significant upregulation of CD4^+^FoxP3^+^ cells in organs from AP + Treg animals in comparison to the AP group. Decidual Treg levels were also augmented. As expected^19^, Treg transfer resulted in a reduction of the fetal death rate. When analyzing surviving fetuses and placentas, we observed that Treg transfer was able to normalize both, placenta size and the shaping of SAs. Interestingly, Treg transfer into AP mice was accompanied by a simultaneous increase of MC numbers in particular of uMCs as well as increased transcript levels of MC-specific protease 1. This strongly suggests a Treg-induced upregulation and activation of MCs at the feto-maternal interface. Furthermore, the blockage of IL-10, the major Treg mediator in this model^30^, hindered Mcpt1 augmentation. Evidence of possible Treg-MC interaction can be found in different models in the literature. For example, Lu and colleagues postulated in 2006 that MCs are essential intermediaries in Treg-dependent tolerance, where IL-9 represents the functional link between the two immune cell populations^20^. This is in agreement with findings reported by Eller and colleagues that suggest that IL-9 production by Tregs recruits and activates MCs^35^. The augmentation in MC numbers after transfer of Tregs in AP mice was not linked to increased IL-9 at the feto-maternal interface. In our model the transfer of Treg was rather associated with an increase of IL-3 and mSCF at the feto-maternal interface. As both are proposed as MC growth factors^36^,^37^,^38^, it is tempting to speculate that Treg act by augmenting these two mediators. In the subsequent studies, we will concentrate on dissecting the factors that are involved in the interaction between these two cell types; this is currently not possible due to the technical limitations.

A crucial event in fetal development is a correct placentation. Defects in placentation can cause miscarriage or pregnancy diseases like pre-eclampsia or IUGR^39^. Here, we found that placentas from DBA/2J-mated CBA/J females are decreased in size as compared to NP BALB/c-mated CBA/J females. This is in agreement with reports claiming that the CBA/J × DBA/2J model represents a valid IUGR model as fetuses grow slower in this combination compared to the CBA/J × BALB/c controls^34^,^40^. Our data show that Treg transfer was able to normalize placenta size. This in turn correlated with an augmentation of the numbers of uMCs. Several studies have linked MCs with placentation^6^,^41^,^42^,^43^ but as of yet no studies have shown that Tregs are relevant in this context. In contrast, several previous reports indicate that Tregs are relevant for implantation^17^,^44^,^45^ and for maintaining immune tolerance^30^,^46^. Our results suggest that Tregs promote the expansion of uMCs, which in turn positively influences placentation.

To ensure an adequate supply of the fetus with nutrients and oxygen, enough maternal blood has to flow to the fetal side. Here, SAs overtake a central role; as pregnancy advances they change their shape to turn into thin-walled venous-like vessels. This process is called SA remodeling and is induced by the selective reduction of the SM layer^11^. Insufficient remodeling is linked to pre-eclampsia, fetal growth restriction, miscarriage and preterm birth^2^,^47^,^48^,^49^. We observed insufficiently remodeled SAs in AP mice; this is indicated by an augmented wall thickness and an increased wall-to-lumen ratio when compared to the NP mice. Immunohistochemically, we found a thicker SM actin layer that indicates a poor apoptosis of this layer. Interestingly, the transfer of Tregs, associated with uMC expansion could normalize both wall thickness and wall to lumen ratio. The SM actin layer was comparable to NP mice. Usually, uMCs are located close to vessels^6^ as we could confirm here. Functionally, uMCs are able to modulate a proper SA remodeling as we reported for MC-deficient Kit^W-sh/W-sh^ animals that were reconstituted with MCs^6^. It was reported that animals depleted of Tregs during pregnancy showed insufficiently remodeled SAs^18^. Whether this was a sole effect of Treg absence or depended on other cells was not further explored in the mentioned study. We propose that the transfer of Tregs into AP mice promotes the expansion of uMCs and they in turn promote the remodeling of SAs.

A successful pregnancy depends on a well-balanced angiogenesis/vasculogenesis system. This is mediated by a broad array of angiogenic factors including the interaction between vascular endothelial growth factor (VEGF) and its receptor VEGFR. Elevated levels of the soluble form of VEGFR, namely sFlt-1, are associated with pregnancy complications including pre-eclampsia^12^. This is due to the fact that sFlt-1 inhibits the mandatory VEGF response by forming a complex with VEGF in the circulation^50^. In the presence of elevated sFlt-1, the positive angiogenic influence of VEGF at the fetal-maternal interface on placental development and SA formation is impaired. Here, we showed that AP mice, which have impaired placentation and insufficient SA remodeling, exhibit significant elevated sFlt-1 levels. In line with this, a study from 2006 demonstrated significantly decreased free VEGF levels in AP mice in contrast to NP mice^34^. The same could be shown for sFlt-1^40^. The adoptive transfer of Tregs and the subsequent upregulation of MCs at the feto-maternal interface are associated with downregulated levels of sFlt-1; this may explain why placentation is restored, SAs properly remodeled, and fewer fetuses died.

The renin-angiotensin-aldosterone pathway is one of the most important blood pressure-regulating systems of the body. Angiotensin (Ang) II is generated from AngI through the activity of angiotensin-converting enzyme (ACE) and involved in vascular adaptions and diseases^51^. Several studies have demonstrated that MC chymase is able to generate AngII from AngI independently of ACE^52^,^53^. Girardi and colleges reported that AP mice show a higher sensitivity to AngII as AngII treatment in DBA/2J-mated CBA/J females lead to a significant change in blood pressure. Without application of AngII there were no differences between the groups. These observations suggest that the local systems of the AP model may serve to increase the effects of circulating AngII by upregulating the AngII receptor^34^. We found that AP mice present upregulated levels of AngII receptor. Transfer of Treg and increase of uMCs was related with downregulation of AngII receptor to the levels observed in NP mice, suggesting that Treg or uMCs secrete molecules that control the expression of the receptor. Upregulation of the AngII receptor at the fetal-maternal interface of pregnant females from the CBA/J × DBA/2J combination may maintain the signal strength of AngII – AngII receptor as compensation for lower AngII concentrations due to decreased MC levels. The chymase AngII generating system is different from the ACE-dependent AngII generating system. Tissue originated renin generates Angiotensinogen I from Angiotensinogen. Generation of AngII from AngI depends on chymase, which is secreted from the granules of MCs. Chymase-dependent AngII does influence tissue remodeling; it is however not involved in the regulation of hypertension^54^. This would explain the insufficient remodeling of SAs in AP mice in the absence of elevated systemic blood pressure. In other models, in which SA remodeling is rather controlled by uNKs, the insufficient remodeling of SA is directly related with maternal hypertension^2^.

The data obtained in our study unravel a so far unsuspected cellular interaction that contributes to pregnancy maintenance. We show that the adoptive transfer of Tregs to animals that reject their fetuses is accompanied by an augmentation of the number of uMCs while having positive effects on placentation and the remodeling of SAs, all together leading to healthy fetuses. The mechanisms behind the positive effect of Treg and MC crosstalk at the fetal maternal interface include the maintenance of angiogenesis balance, especially the normalization of sFlt-1 as well as the modulation of receptors for angiotensin. Our study reveals a further aspect of Treg action during pregnancy and highlights the interaction between the innate and adaptive immune system with the final goal of supporting pregnancy and fetal growth.

## Materials and Methods

### Animals

The present study was approved by the German Ministry (203.h-42502-2-868 Magdeburg, Landesverwaltungsamt, Halle) and carried out according to institutional guidelines and the Guide for the Care and Use of Agricultural Animals in Agricultural Research and Teaching, USA.

The stage of the estrous cycle of sexually mature CBA/J females was determined based on the typical cell content of the fresh vaginal lavage with 0.9% sodium chloride. Females were sacrificed when the stage was clearly defined. To confirm the stage, samples were stained with hematoxylin/eosin (H/E) and analyzed by light microscopy (Axio Observer.A1, Zeiss; magnification ×200).

Female CBA/J (H2^k^), male DBA/2J (H2^d^) and male BALB/c (H2^d^) mice were purchased from Charles River (Germany) and Janvier (France). Animals were maintained in the institute’s own barrier animal facility with a light/dark cycle of 12/12 h. Female CBA/J mice were mated either with BALB/c males (normal pregnant, NP) or DBA/2J males (abortion-prone, AP). Successful mating was confirmed by the detection of a vaginal plug. The day of plug detection was considered as day 0 of pregnancy and females were separated from the males. CBA/J females were sacrificed on days 0, 2, 5 or 12 of pregnancy. The number of animals included in each group and of samples used for each analysis is depicted in the Figure legends.

### Isolation and Transfer of Tregs

Tregs were isolated from the spleens and lymph nodes (inguinal, para-aortic) of normal pregnant females on day 14 of pregnancy as described elsewhere^19^,^30^. 2 × 10^5^ cells were diluted in 200 μl PBS and injected intravenously into DBA/2J-mated CBA/J females at day 0 of pregnancy. Control animals received PBS. In a further experiment, we used samples from animals that have received Treg following the same protocol and were additionally treated with 1 mg of anti-IL-10 mAb at days 0 and 7 of pregnancy^30^. Controls received either Treg alone or Treg + 1 mg IgG^30^. The abortion rates in the groups was as follows: median of abortion: 0.00 for AP + Treg; 10.55 for AP+IgG+Treg and 33.31 for AP + anti-IL-10 + Treg.

### Sample collection and histology

Uterine horns from animals at different phases of the estrous cycle, or gd 0, 2 or 5 were dissected, fixed in 96% ethanol, embedded in paraffin and cut longitudinally into 5 μm sections. For visualization of uMCs, sections were stained with Toluidine blue dye (0.1% aqueous solution). The number of uMCs was calculated per 1 mm^2^ by using an Eyepiece-micrometer (Zeiss).

One implantation site per female was collected on day 12 of pregnancy for paraffin embedding with previous fixation in 4% (w/v) PFA with 0.1 M sucrose (pH 7.4) for 6 h. Placental and decidual tissue were washed with PBS, pH 7.40, snap-frozen, and kept at −80 °C until use for RNA and protein isolation.

### Quantitative histological measurements

The measurements of placental surface areas were performed according to Croy and colleagues^55^. Briefly, 5 μm transversal cross sections of feto-placental units at day 12 of pregnancy were stained with H/E and the software AxioVision4 (Zeiss) was used to measure the placental surface areas at a X10 magnification. The quantitative measurements of SAs were performed as described elsewhere^53^. Shortly, the SA wall and lumen diameters were measured and/or expressed as wall to lumen ratios and wall thickness.

### Immunofluorescence

Heat induced antigen retrieval of transversal cross-sections of feto-placental units was carried out in 10 mmol/l Tris/1mmol/l EDTA buffer (pH 6.0) for 10 min in the microwave. Incubation with anti-smooth muscle actin (Dako) was done overnight at 4 °C followed by an AF555 conjugated secondary antibody (Invitrogen) for 1 h at room temperature. VECTASHIELD mounting medium containing DAPI (VECTOR laboratories) was used to counterstain DNA.

### Flow Cytometry

Tissue from spleen, thymus, lymph nodes (inguinal, mesenteric and para-aortic) and oviducts were processed as described elsewhere^19^. Uterine tissue was enzymatically digested using Liberase TL (Roche). Cells were stained with the following antibodies: FITC-conjugated anti-FcεRIα, PE-conjugated anti-CD117, PerCP-Cy^TM^5.5-conjugated anti-CD4, Alexa Fluor 647-conjugated anti-Foxp3. Besides the FcεRIα antibody (eBioscience) all antibodies were purchased from Becton Dickinson (BD).

### Real Time Reverse Transcriptase Polymerase Chain Reaction (RT-PCR)

Total RNA isolation from frozen decidual tissue was performed by using Trizol (Gibco) and a homogenizer (Ultra Turrax T8). The RNA was extracted with chloroform, precipitated with isopropanol, washed in 80% ethanol, and finally diluted in RNase-free water. RNA quantity and quality was determined by ultraviolet absorbance at 260 nm. Total RNA (2 μg) was initially incubated with oligo dTs (Amersham) for 10 min at 75 °C followed by 5 min incubation on ice. Subtracted mRNA was than incubated with dNTPs (2.5 mmol/L, Amersham), DNase I (2 U/ml, Stratagene) and RNase inhibitor (40 U/ml, Promega) in reaction buffer. The mixture was incubated for 30 min at 37 °C and afterwards heated for 5 min at 75 °C. Reverse transcriptase (200 U/ml, Promega) and RNase inhibitor in RNase-free water was added to the mixture and incubated at 42 °C for 60 min. Incubation at 94 °C for 5 min followed.

Real-time polymerase chain reaction (RT-PCR) amplifications were performed in an iCycler (BioRad). Beta-actin or GAPDH were used as housekeeping genes. Experiments were run in duplets. Amplification reactions were performed as follows: initial denaturation at 95 °C for 5 min followed by 40 cycles of denaturation at 95 °C for 45 sec and annealing at either 56 °C or 60 °C for 60 sec.

### SDS page, Western blot

During and after sonication placental tissue was incubated in lysis buffer (10% NP-40, 0.1 mg/ml n-Dodecil-β-D-maltoside, 500 mM sodium fluoride, 10 mM Sodium metavanadate, 100 mM PMSF, 1M Tris, 0.5 M EDTA, 5 M NaCl) for 60 min. Afterwards cells were centrifuged, supernatants were collected and stored overnight at −80 °C. Protein content was determined by Bradford assay (BioRad) as indicated by the manufacturers. Proteins (50 μg) were resolved on a 8% or 12% SDS-PAGE at 100 V and transferred onto a 0.45 μm nitrocellulose membrane in transfer buffer containing 20%(v/v) methanol, 0.19 M glycine and 0.025 M Tris (pH 8.3) at a constant voltage on ice. After blocking nonspecific-binding sites with 5% (w/v) skim milk powder in TBS with 0.05% (v/v) Tween for 1 h, blots were incubated using the following antibodies: Flt-1 (C-17, 1:250, Santa Cruz), AT_2_ (H-143, 1:500, Santa Cruz), β-actin (AC-15, 1:10000, Sigma-Aldrich), GAPDH (FL-335, 1:10000, Santa Cruz). The expression of sFlt-1 (two relevant isoforms for pregnancy) was analyzed by using the program GeneTools (SynGene) and was referred to β-actin. The expression of AT_2_ (41 kDa) was referred to GAPDH.

### Data analysis and statistics

GraphPad Prism 5.0 software was used for statistical analysis. The Shapiro-Wilk test was applied to determine whether or not the values follow a normal distribution. Data obtained by flow cytometry and PCR among all groups were calculated by the non-parametric Kruskal-Wallis test followed by the Mann-Whitney-*U* test to calculate the difference between two independent groups. Quantification of uterine MCs per 1 mm^2^ was done by calculating the mean MC number at different areas of the uterus. Groups were compared by the unpaired *t*-test. For histological measurements, two to ten SAs per female were measured by AxioVision4 (Zeiss), the mean was calculated, and the differences between the animal groups analyzed using unpaired *t*-test; differences regarding the placental surface areas between the groups (1 to 2 placentas/female/group) were analyzed by Mann-Whitney-*U* test. For all tests, *P* value < 0.054 was considered to be statistically significant. The number of animals or samples used for a determined experiment as well as the statistical test used and the *P* values obtained are indicated in each Figure legend.

## Additional Information

**How to cite this article**: Woidacki, K. *et al.* Transfer of regulatory T cells into abortion-prone mice promotes the expansion of uterine mast cells and normalizes early pregnancy angiogenesis. *Sci. Rep.* **5**, 13938; doi: 10.1038/srep13938 (2015).

## Supplementary Material

Supplementary Information
