# Supplementary material for: Transfer of regulatory T cells into abortion-prone mice promotes the expansion of uterine mast cells and normalizes early pregnancy angiogenesis
Source: Sci Rep. 2015 Sep 10;5:13938. doi: 10.1038/srep13938 (PMC4565045; doi:10.1038/srep13938)
Supplement: Supplementary Information [file srep13938-s1.pdf]

Supplementary information

Transfer of regulatory T cells into abortion-prone mice promotes the expansion of uterine mast cells and normalizes early pregnancy angiogenesis

Katja Woidacki[1,2], Nicole Meyer[1,2], Anne Schumacher[2], Alexandra Goldschmidt[2], Marcus Maurer[3], Ana Claudia Zenclussen[2]

[1] These authors contributed equally to this work

[2] Experimental Obstetrics and Gynecology, Medical Faculty, Otto-von-Guericke University, Magdeburg, Germany.

[3] Department of Dermatology and Allergy, Charité – Universitätsmedizin Berlin, Germany.

*: Corresponding author: Ana C. Zenclussen, Experimental Obstetrics and Gynecology, Medical Faculty, Otto-von-Guericke University, Gerhart-Hauptmann Str. 35, 39108 Magdeburg, Germany. Phone: +49 391 6717460, Fax: +49 391 6717440, E-mail: ana.zenclussen@med.ovgu.de

Running title: Tregs contribute to angiogenesis through uMCs

Legends to supplementary Figures

Supplementary Figure 1: Transfer of Tregs resulted in augmented expression of IL-3 and mSCF but not of IL-9 at the feto-maternal interface.

Normal pregnant (NP, n=6), abortion-prone (AP, n=6-7) and abortion-prone animals transferred with Tregs (AP+Treg, n=6-9) were sacrificed at day 12 of pregnancy. IL-3, IL-9 and mSCF mRNA levels were analyzed by real time PCR and the data expressed as $^{2\text{-}\Delta cT}$. Data are shown as single values with medians. Statistically significant values are indicated as follows: *p<0.050, **p<0.005 (Mann Whitney-*U* test).

Supplementary Figure 2: Blockage of IL-10 hampered the augmentation of Mcpt-1 in mice that have been transferred with Tregs

Abortion-prone mice treated with Treg (AP + Treg, n=7), abortion-prone mice treated with Treg and 1 mg IgG (AP + IgG + Treg, n=4) as well as abortion-prone animals transferred with Tregs and treated with 1 mg anti-IL-10 at days 0 and 7 of pregnancy (AP+Treg, n=6-9) were sacrificed at day 12 of pregnancy. Mcpt-1 mRNA levels were analyzed by real time PCR and the data expressed as $^{2\text{-}\Delta cT}$. Data are shown as single values with medians. Statistically significant values are indicated as follows: *p<0.050, **p<0.005 (Mann Whitney-*U* test).

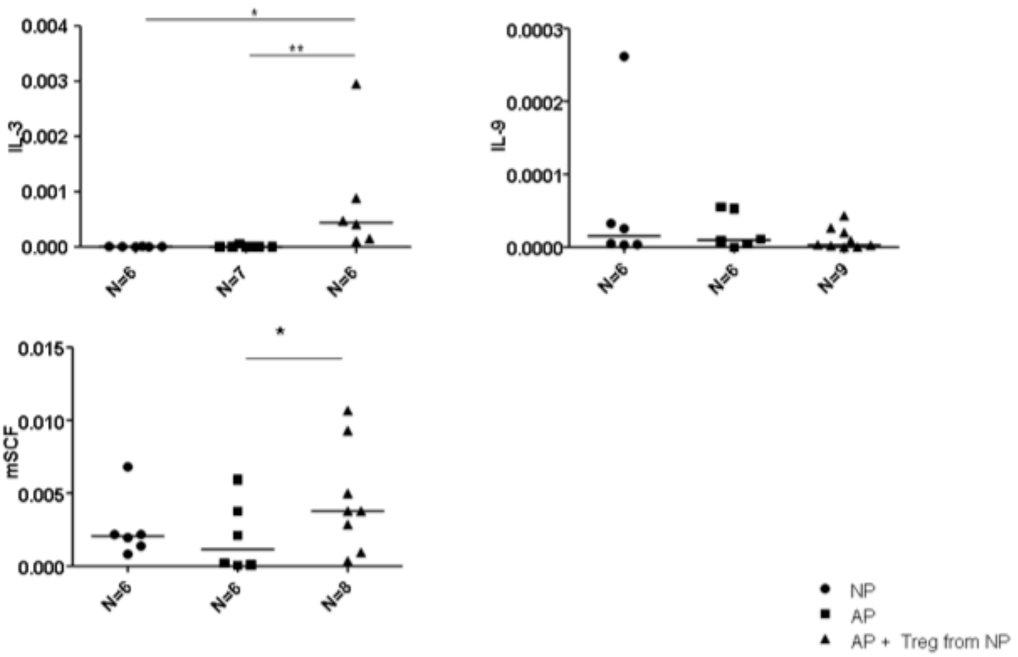

*
**
0.004
0.003
0.002
0.001
0.000
IL-3
N=6
N=7
N=6
0.0003
0.0002
0.0001
0.0000
IL-9
N=6
N=6
N=9
*
0.015
0.010
0.005
0.000
mSCF
N=6
N=6
N=8
NP
AP
AP + Treg from NP

Suppl. Fig. 1

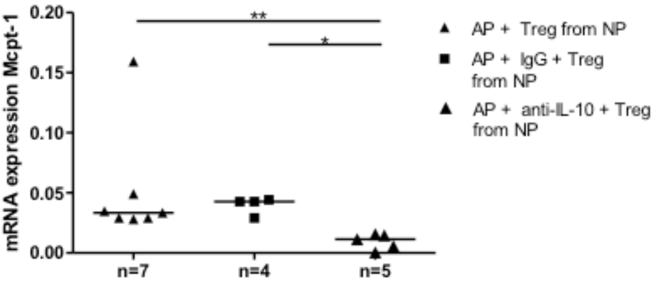
mRNA expression Mcpt-1
0.20
0.15
0.10
0.05
0.00
**
*
n=7
n=4
n=5
AP + Treg from NP
AP + IgG + Treg from NP
AP + anti-IL-10 + Treg from NP

Suppl. Fig. 2
